# Rapid Evolution of Multidrug Resistance in a Candida lusitaniae Infection during Micafungin Monotherapy

**DOI:** 10.1128/aac.00543-23

**Published:** 2023-07-10

**Authors:** Nancy E. Scott, Serin Edwin Erayil, Susan E. Kline, Anna Selmecki

**Affiliations:** a University of Minnesota, Bioinformatics and Computational Biology Program, Minneapolis, Minnesota, USA; b University of Minnesota, Department of Microbiology and Immunology, Minneapolis, Minnesota, USA; c University of Minnesota Medical School, Department of Medicine, Division of Infectious Diseases and International Medicine, Minneapolis, Minnesota, USA

**Keywords:** *Candida lusitaniae*, antifungal drug resistance, echinocandin, FKS1, ERG3

## Abstract

Candida (Clavispora) lusitaniae is a rare, emerging non-*albicans Candida* species that can cause life-threatening invasive infections, spread within hospital settings, and rapidly acquire antifungal drug resistance, including multidrug resistance. The frequency and spectrum of mutations causing antifungal drug resistance in C. lusitaniae are poorly understood. Analyses of serial clinical isolates of any Candida species are uncommon and often analyze a limited number of samples collected over months of antifungal therapy with multiple drug classes, limiting the ability to understand relationships between drug classes and specific mutations. Here, we performed comparative genomic and phenotypic analysis of 20 serial C. lusitaniae bloodstream isolates collected daily from an individual patient treated with micafungin monotherapy during a single 11-day hospital admission. We identified isolates with decreased micafungin susceptibility 4 days after initiation of antifungal therapy and a single isolate with increased cross-resistance to micafungin and fluconazole, despite no history of azole therapy in this patient. Only 14 unique single nucleotide polymorphisms (SNPs) were identified between all 20 samples, including three different *FKS1* alleles among isolates with decreased micafungin susceptibility and an *ERG3* missense mutation found only in the isolate with increased cross-resistance to both micafungin and fluconazole. This is the first clinical evidence of an *ERG3* mutation in C. lusitaniae that occurred during echinocandin monotherapy and is associated with cross-resistance to multiple drug classes. Overall, the evolution of multidrug resistance in C. lusitaniae is rapid and can emerge during treatment with only first-line antifungal therapy.

## INTRODUCTION

Candida (Clavispora) lusitaniae is a rare and understudied opportunistic fungal pathogen. C. lusitaniae can cause fatal infections in immunocompromised individuals (1), has been implicated in nosocomial transmission events (2, 3), and is known for its ability to acquire antifungal drug resistance within days of treatment (4). Early studies of antifungal drug resistance in C. lusitaniae clinical isolates identified increases in resistance in as little as 3 days during combination therapy (5) and within 9 days during amphotericin B therapy (6). Multidrug resistance has also been reported in C. lusitaniae (7), which is particularly notable in light of its close relationship to Candida auris, a recently emerged, multidrug-resistant pathogen that has spread rapidly around the world (8). Little is known about the frequency and mechanisms of mutations underlying drug resistance in C. lusitaniae or about the order and spectrum of mutations underlying emergence of multidrug resistance in patients during therapy.

Few therapeutic options exist for treatment of invasive Candida infections. Major antifungal drug classes are limited to echinocandins, including micafungin, azoles, including fluconazole, and polyenes, including amphotericin B. Echinocandins inhibit 1,3-β-d-glucan synthase, and most echinocandin resistance in Candida species is caused by mutations in *FKS1*, which encodes the catalytic subunit of this enzyme (9, 10). Azoles target Erg11, part of the ergosterol biosynthesis pathway, leading to accumulation of 14α-methylfecosterol, which is converted to toxic 14α-methyl-3,6-diol by Erg3 (11). Mutations of genes encoding proteins involved in ergosterol biosynthesis are a common cause of azole resistance in Candida species. Polyenes target ergosterol in the fungal cell membrane and extract it from the lipid bilayer (12). Mutations resulting in altered sterol composition, including ergosterol biosynthesis mutations, can lead to resistance and have been reported in Candida albicans, C. lusitaniae, and Candida tropicalis (13–16). Resistance to more than one of these three classes of drug, i.e., multidrug resistance, does occur and is more common in haploid species, including C. lusitaniae, C. auris, and Candida glabrata, than in the diploid C. albicans (9, 17). Typically, multidrug resistance in Candida species is due to the accumulation of multiple distinct resistance mutations; however, the order and frequency in which these mutations occur, especially within patients undergoing antifungal therapy, is poorly understood.

To determine the spectrum and effects of mutations that arise during acute antifungal monotherapy, we analyzed 20 serial C. lusitaniae bloodstream isolates collected daily from an individual patient receiving micafungin monotherapy for 11 days during a single hospital admission. We performed comparative phenotypic and genomic analysis on all 20 serial isolates. Increased micafungin MIC values occurred within 4 days of initiating treatment. Surprisingly, cross-resistance to fluconazole arose simultaneously despite no history of azole treatment in this patient (going back 9 years). Comparative analysis of whole-genome sequencing (WGS) data identified no more than five single nucleotide polymorphisms (SNPs) between any two isolates, confirming that all isolates were clonally related. Six isolates with decreased micafungin susceptibility carried one of three unique *FKS1* missense mutations, suggesting within-patient clonal interference of drug-resistant sublineages. Strikingly, one isolate acquired increased cross-resistance to both micafungin and fluconazole, despite no prior patient exposure to azole drugs, and this phenotype was associated with an *ERG3* missense mutation. To our knowledge, this is the first report of clinical occurrence of C. lusitaniae fluconazole cross-resistance arising during echinocandin monotherapy.

## RESULTS

### Patient history.

A 50-year-old man with a complicated medical history, including primary sclerosing cholangitis, failed liver transplant, chronic kidney disease, and portal vein thrombosis, underwent attempted portal vein recanalization. Treatment was complicated by a transplenic puncture, and he was admitted for a gastrointestinal bleed, hypovolemic shock, and acidosis. He was discharged to an acute rehabilitation center but was readmitted soon after with fevers, groin pain, and progressive kidney and liver disease. He was found to have sepsis due to C. lusitaniae fungemia. Immunosuppression was reduced. On the second day of admission, he was started on intravenous micafungin at 150 mg every 24 h. Azoles were avoided due to the patient’s elevated bilirubin. He had no known azole use during this admission or in the past, based on available clinical records covering a 9-year history.

**FIG 1:**
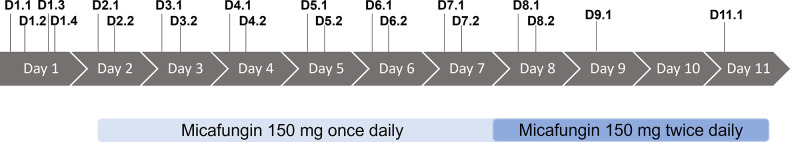
Timeline of hospital admission, clinical isolate collection, and micafungin therapy. All isolates are single-colony cultures originating from individual blood culture specimens. Isolates are numbered by day of admission and order of specimens (e.g., D1.2 is day 1, isolate number 2). See Table S1 for isolate details.

The patient required placement of a hemodialysis catheter and a peripherally inserted central catheter (PICC) line. In addition to the original portal vein thrombus, he developed an occlusive thrombus in his right arm cephalic vein. On day 8, his micafungin dose was increased to 150 mg every 12 h to maintain therapeutic levels in the setting of a new active gastrointestinal bleed. Blood cultures remained positive for C. lusitaniae for the duration of admission (11 days). The patient was not a good candidate for interventional radiology treatment of the clots, his invasive lines could not be removed, and all of these were thought likely to be sources of persistent candidemia. A care conference was held, and the patient and his family elected for hospice. He was transitioned to comfort care on day 10 of hospital admission and passed away on day 11. Figure 1 depicts a timeline of antifungal therapy and collection of blood culture isolates. Isolates analyzed for this study are single-colony subcultures from individual blood cultures and are numbered by day of admission (D1 to D11) and order of collection per day (e.g., D1.2 is the day 1, isolate number 2; see details in Table S1 in the supplemental material).

### Antifungal drug resistance, including azole cross-resistance, develops within days under micafungin monotherapy.

**FIG 2:**
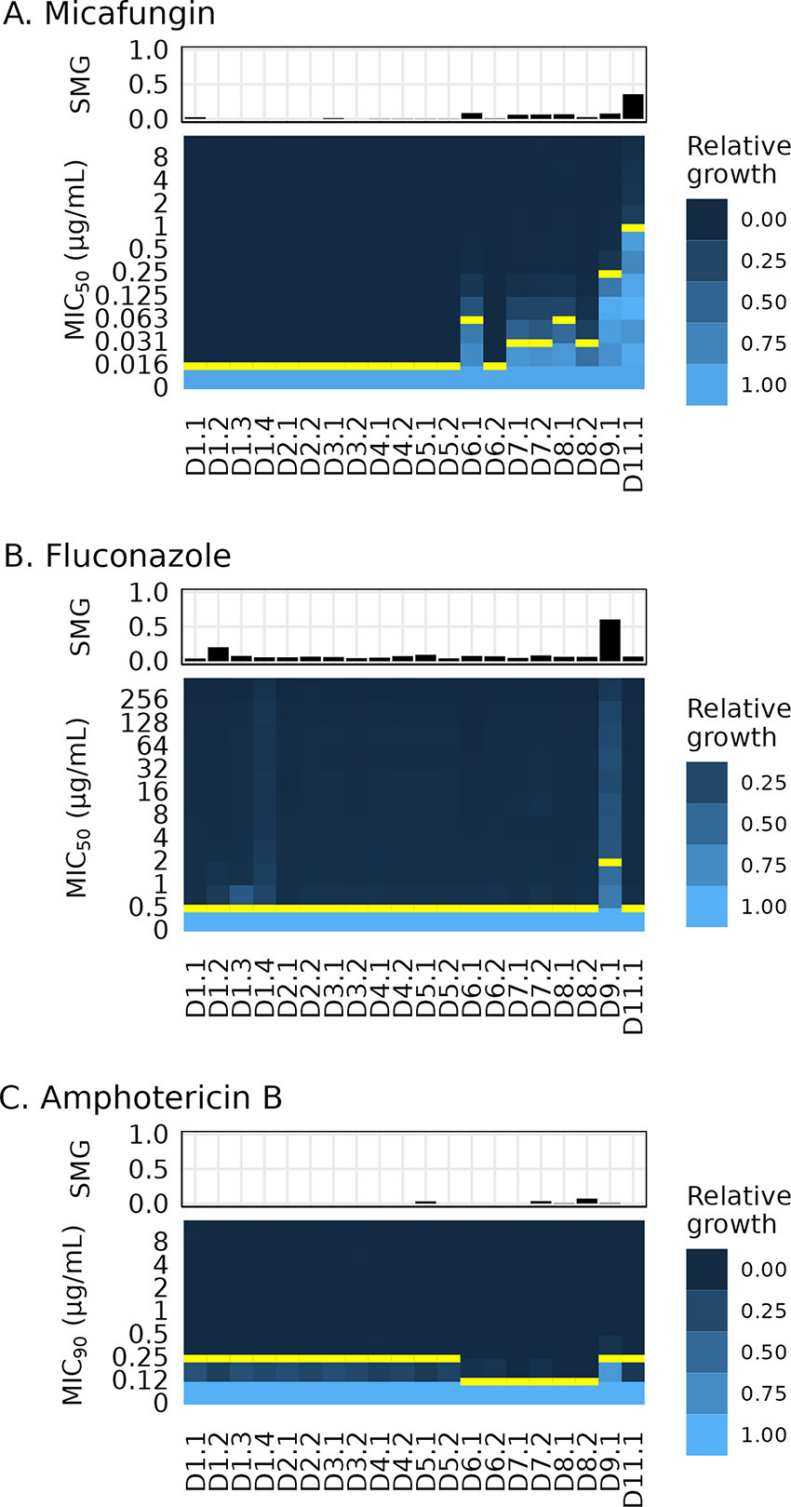
Decreased drug susceptibility and tolerance appeared within 9 days of echinocandin monotherapy during C. lusitaniae infection. Heatmaps of relative growth at increasing concentrations of antifungal drug (MICs) and bar charts of tolerance (supra-MIC growth [SMG]) for three drug classes. The MIC is marked with a yellow bar for each isolate. MIC_50_ is the lowest concentration of drug that decreased the 24-h growth to less than 0.5 of the growth of the no-drug control, and MIC_90_ is the lowest concentration of drug that decreased the 24-h growth to less than 0.9 of the growth of the no-drug control (see Materials and Methods). SMG is the proportion of growth at 48 h in all wells with drug concentrations above the MIC relative to the growth of the no-drug control. (A) Micafungin (MCF) SMG and MIC_50_. (B) Fluconazole (FLC) SMG and MIC_50_. (C) Amphotericin B (AMB) SMG and MIC_90_. Each plot represents the average values from 3 independent assays.

During the patient’s hospitalization, clinical antifungal susceptibility testing of only the initial C. lusitaniae bloodstream isolate (D1.1) was performed. All MIC values for this isolate (fluconazole, 0.25 μg/mL, voriconazole, ≤0.008 μg/mL, micafungin, ≤0.03 μg/mL, and amphotericin B, 0.12 μg/mL) were below the established Clinical and Laboratory Standards Institute epidemiologic cutoff values for C. lusitaniae (18). To determine if drug sensitivity decreased during micafungin therapy, we performed MIC testing by broth microdilution of all 20 serial candidemia isolates. We quantified the MICs to the three major drug classes (micafungin, fluconazole, and amphotericin B) at 24 h. Starting at day 6, seven isolates had increased micafungin MIC values (D6.1, D7.1, D7.2, D8.1, D8.2, D9.1, and D11.1), with the highest MIC observed in the last isolate (D11.1)—a 64-fold increase to 1 μg/mL micafungin (Fig. 2A; Tables S1 and S2). Notably, a single isolate (D9.1) exhibited a 4-fold increase in fluconazole MIC (from 0.5 to 2 μg/mL FLC) in addition to a 16-fold increase in the micafungin MIC, to 0.25 μg/mL (Fig. 2A and B; Tables S1 and S2). All isolates were sensitive to amphotericin B (Fig. 2C; Tables S1 and S2).

We next quantified the antifungal drug tolerance for each isolate. Antifungal drug tolerance is residual growth of an isolate in drug concentrations above its MIC and has been associated with persistent and recurrent clinical C. albicans infections and treatment failure in animal models of C. albicans and C. glabrata infection (19, 20). While most drug tolerance literature has focused on fluconazole, tolerance of polyenes and echinocandins has been reported (20). To identify increases in drug tolerance during micafungin therapy, we determined the supra-MIC growth (SMG) at 48 h for all isolates for micafungin, fluconazole, and amphotericin B, calculated as the mean of all growth in wells with drug concentrations above the 24-h MIC value normalized to the growth of the no-drug control (the maximum possible SMG value is 1.0; see Materials and Methods). Isolate D11.1 demonstrated increased micafungin tolerance with an SMG value of 0.36 (Fig. 2A; Table S1). Strikingly, isolate D9.1 acquired remarkable tolerance of fluconazole, with an SMG value of 0.60 (Fig. 2B; Table S1), which is greater than the mean SMG of 0.4 reported in C. albicans isolates from persistent candidemia cases (20). No isolates were tolerant of amphotericin B (Fig. 2C; Table S1). In summary, isolates with decreased susceptibility and increased tolerance of micafungin were observed between 6 and 11 days after initiation of micafungin monotherapy and one isolate had increased resistance to both micafungin and fluconazole, as well as fluconazole tolerance, in 9 days.

### Fitness costs are varied for isolates with decreased susceptibility in the absence of drug.

**FIG 3:**
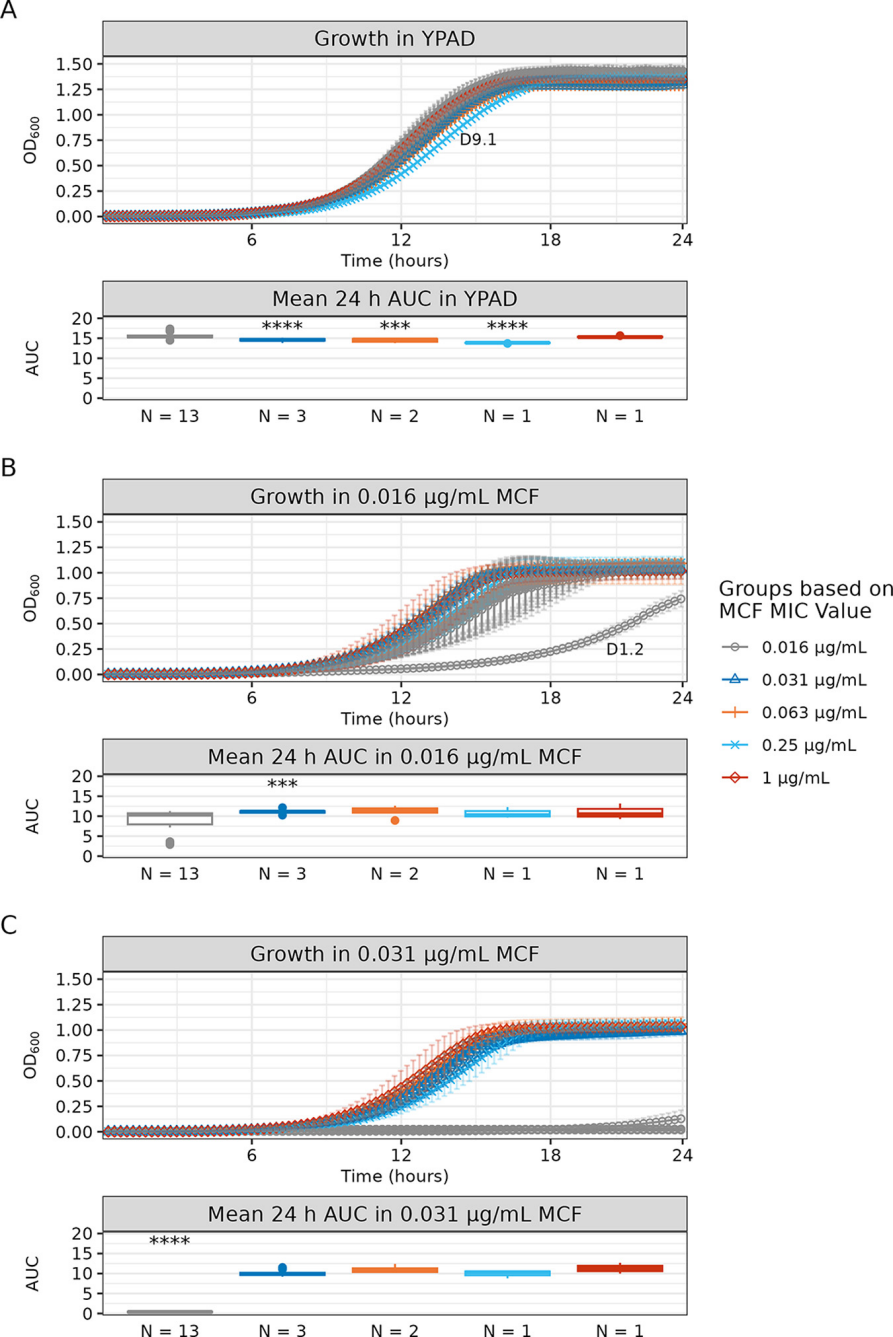
The isolate with the greatest increase in the micafungin MIC value did not have a significant fitness cost relative to the fitness of drug-sensitive isolates. Shown are the 24-h growth curve and a box plot of the mean 24-h area under the logistic curve (AUC-L) values for all serial isolates in the absence and presence of micafungin (MCF). Isolates are grouped by MCF MIC results. The number of isolates per group is indicated on the *x* axis of each box plot. (A) Growth and mean AUC-L values in YPAD medium. (B) Growth and mean AUC-L values in 0.016 μg/mL MCF. A single isolate (D1.2) did not reach stationary phase and is labeled on the growth curve. (C) Growth and mean AUC-L values in 0.031 μg/mL MCF. Groups with any statistically significant difference in pairwise comparison of mean values are marked by asterisks in the box plot (***, *P* ≤ 0.001; ****, *P* ≤ 0.0001; see Table S3 for all statistical comparisons). Growth curves were performed in triplicate; mean slope values and standard deviations are shown. See Table S3 for growth curve summary statistics, Welch’s ANOVA omnibus testing of group mean AUC-L values, and Games-Howell *post hoc* pairwise testing.

Mutations that confer advantages like drug resistance can be detrimental in other environments. To compare the fitness of isolates in the absence and presence of drug, we performed 24-h growth curve analyses in yeast extract-peptone-adenine-dextrose (YPAD) medium without drug and with two low concentrations of micafungin (0.016 μg/mL and 0.031 μg/mL). We performed statistical analysis of the area under the logistic curve (AUC-L) and grouped isolates by their micafungin MIC values for comparison (Fig. 3; Table S3). AUC-L is a single value that integrates information from growth rate, doubling time, and carrying capacity (21).

In the absence of drug, there was a slight decrease in AUC-L among isolates with increased micafungin MIC values, which reached statistical significance for the six isolates with micafungin MICs of 0.031, 0.063, and 0.25 μg/mL. Notably, the isolate with the highest micafungin MIC value (D11.1, MIC = 1 μg/mL) had an AUC-L similar to those of micafungin-sensitive isolates, indicating that resistance in this isolate did not correlate with a fitness cost (Fig. 3A). In contrast, the isolate with the second highest micafungin MIC value (D9.1, MIC = 0.25 μg/mL) had the longest doubling time of all the isolates in the absence of drug, but its carrying capacity was greater than those of the other isolates with increased micafungin MIC values, indicating that D9.1 had growth kinetics that were distinct from those of the other micafungin-resistant isolates (Fig. 3A; Table S3).

Next, we compared the AUC-L values in the presence of micafungin. At 0.016 μg/mL micafungin, most of the micafungin-sensitive isolates exhibited various decreases in AUC-L, with a single isolate (D1.2) having a large decrease in fitness relative to those of all other isolates (Fig. 3B). At 0.031 μg/mL micafungin, the seven isolates with increased micafungin MIC values had similar AUC-L values that were significantly higher than those of micafungin-sensitive isolates (Fig. 3C). In summary, most of the isolates with decreased micafungin susceptibility had slightly decreased growth relative to the growth of the micafungin-sensitive isolates in the absence of drug; however, the isolate with the greatest increase in micafungin resistance (D11.1) did not incur the same fitness cost, suggesting the acquisition of a refined resistance mechanism.

### All of the C. lusitaniae serial isolates are clonally related.

To determine the genetic mutations underlying these phenotypic differences, we performed comprehensive comparative genomic analyses. The selection of a reference genome can influence analyses like mutation detection and phylogenetic inference (22), so we first evaluated six different genome assemblies to identify an optimal reference genome. We aligned reads of the first clinical isolate (D1.1) to each reference genome assembly and quantified the percentages of mapped reads and properly paired reads (see Table S4 for assembly information and mapping statistics) (23–29). The assembly with the highest percentage of properly paired mapped reads, FDA-ARGOS strain 655 (NCBI accession number GCA_014636115.1), was used as the reference genome for all subsequent analysis (25).

**FIG 4:**
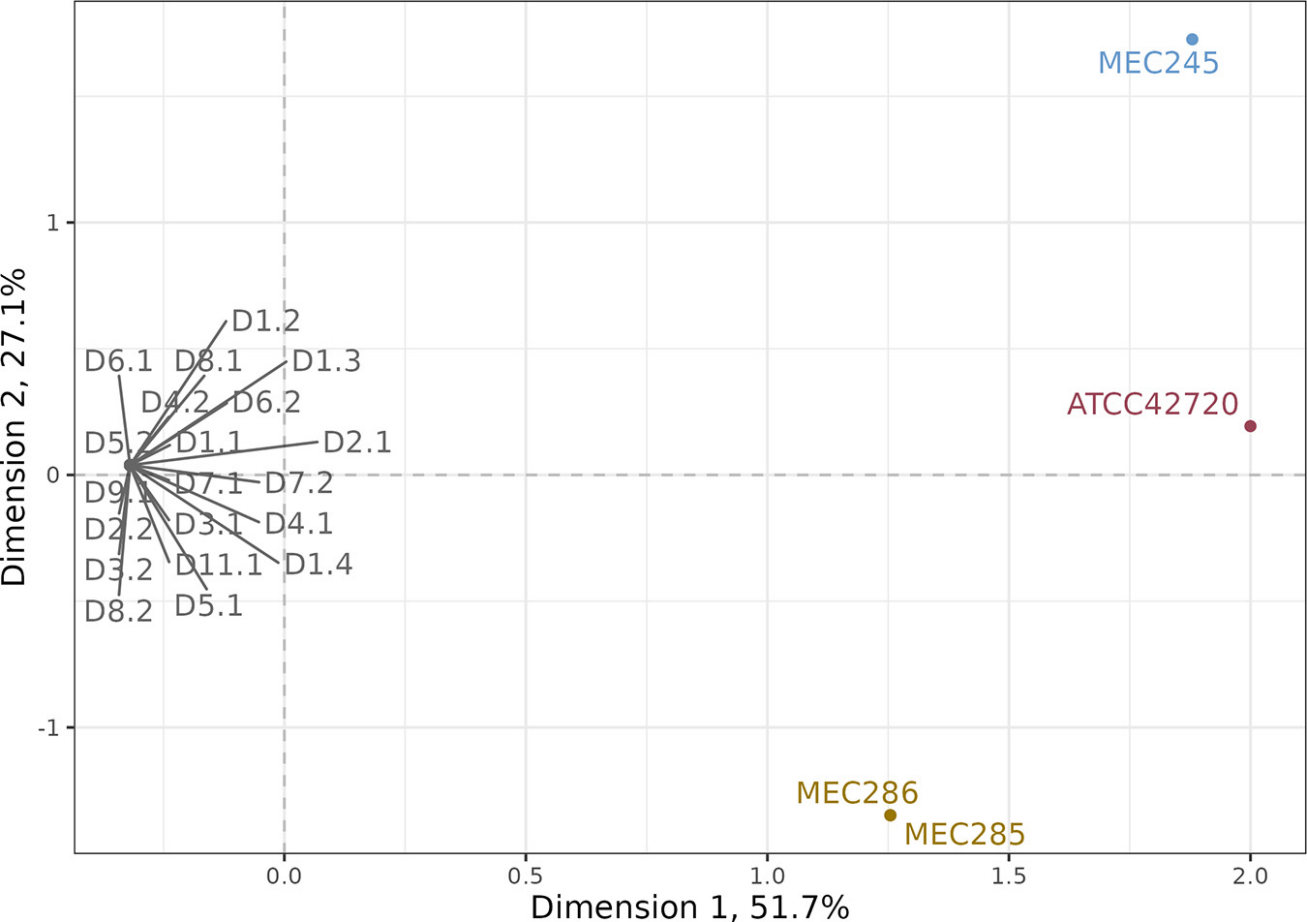
All serial isolates are part of a single clonal group. Scatterplot of multiple correspondence analysis (MCA)-based clustering using genome-wide SNP data from 20 serial isolates (D1.1 to D11.1) and 4 C. lusitaniae isolates from independent sources (MEC245, isolate from the first unrelated candidemia patient; MEC285 and MEC286, isolates from the second unrelated candidemia patient; and ATCC 42720, standard C. lusitaniae reference genome from a clinical isolate). The first two dimensions of the MCA, plotted as the *x* and *y* axes, represent 51.7% and 27.1% of all variation between samples.

We next addressed whether all 20 clinical isolates arose from a common ancestor using comparative analysis of whole-genome-sequencing (WGS) data. We examined the clonal relationship among all 20 serial isolates and 4 additional independent isolates using single nucleotide polymorphism (SNP) data. The unrelated isolates were three C. lusitaniae bloodstream isolates obtained from two different patients in the same metro area (MEC245, MEC285, and MEC286) and the standard C. lusitaniae reference genome clinical strain (ATCC 42720; WGS data deposited in NCBI under accession number GCA_003675505.1 was used) (28). We used SNP data for all 24 samples to perform clustering by multiple correspondence analysis (MCA), a generalization of principal-component analysis for categorical data (30, 31). The first two dimensions of the MCA accounted for >78% of the variation between samples relative to the FDA-ARGOS strain 655 reference genome. As seen by plotting the first two dimensions of the MCA, all 20 serial isolates clustered together, collapsing to a single point that was separated from all samples from independent sources, indicating that the serial isolates were one clonal group (Fig. 4). Finally, analysis of copy number variations (CNVs) identified that the isolates were all euploid, with no chromosomal or segmental chromosome copy number differences between isolates (Fig. S1), further supporting the clonal origin of all 20 isolates.

### Very few point mutations exist between isolates.

After determining that all isolates represented a single clonal group, we examined genetic differences between isolates to identify potential causes of phenotypic variability. We analyzed SNP data to identify isolate-specific mutations—mutations present in one or more isolates but not fixed in all 20 isolates relative to the reference genome. A total of 14 unique, high-confidence, isolate-specific SNPs were identified, including 9 missense, 2 synonymous, and 3 intergenic SNPs (Table 1). Most missense mutations (7 of 9) were found only in a single isolate, while *RLM1^N181K^* (a change of N to K at position 181 encoded in RLM1) was shared by three isolates and *FKS1^L1355S^* was shared by four isolates. Two of the three intergenic SNPs were shared by different subsets of isolates. Two synonymous mutations were identified, each in a single isolate. Pairwise comparison of isolates revealed zero to five SNPs between any two isolates, consistent with a clonal relationship (Table S5). Using the same approach of investigating unfixed mutations and performing pairwise comparisons, we did not identify any high-confidence, isolate-specific indels.

**TABLE 1:** Isolate-specific single nucleotide polymorphisms among all C. lusitaniae isolates relative to the FDA-ARGOS strain 655 reference genome

Isolate	Chromosome no.	Position (bp)	Variant type	Nucleic acid		Alternate frequency	Amino acid change	Gene
				Reference	Alternate			
D1.1	2	1137176	Intergenic	G	A	1.00	NA^*a*^	NA
	5	768831	Synonymous	C	T	1.00	Gly202Gly	*RAD16*
D1.3	5	985590	Missense	C	A	1.00	Asn181Lys	*RLM1*
D1.4	2	1137176	Intergenic	G	A	1.00	NA	NA
D2.2	2	1137176	Intergenic	G	A	1.00	NA	NA
D3.1	2	1137176	Intergenic	G	A	1.00	NA	NA
D3.2	5	985590	Missense	C	A	1.00	Asn181Lys	*RLM1*
	6	489657	Intergenic	C	T	1.00	NA	NA
D4.1	2	1137176	Intergenic	G	A	1.00	NA	NA
	4	234620	Missense	G	T	1.00	Asp340Tyr	*EPL1*
D6.1	2	1255851	Missense	G	A	1.00	Arg1352Cys	*FKS1*
D6.2	2	1137176	Intergenic	G	A	1.00	NA	NA
D7.1	2	897733	Synonymous	G	A	1.00	Thr831Thr	*SEC24*
	2	1255841	Missense	A	G	1.00	Leu1355Ser	*FKS1*
	2	1659448	Intergenic	G	A	1.00	NA	NA
	2	2198841	Missense	G	A	1.00	Glu386Lys	*SYG1*
D7.2	2	1255841	Missense	A	G	1.00	Leu1355Ser	*FKS1*
	2	1659448	Intergenic	G	A	1.00	NA	NA
D8.1	2	1255841	Missense	A	G	1.00	Leu1355Ser	*FKS1*
	2	1659448	Intergenic	G	A	1.00	NA	NA
D8.2	2	1255841	Missense	A	G	1.00	Leu1355Ser	*FKS1*
	2	1659448	Intergenic	G	A	1.00	NA	NA
D9.1	2	1659448	Intergenic	G	A	1.00	NA	NA
	6	252469	Missense	C	A	1.00	Gln308Lys	*ERG3*
D11.1	2	148547	Missense	G	T	1.00	Asp333Glu	*SIP3*
	2	1257850	Missense	C	A	1.00	Leu685Phe	*FKS1*
	3	1493626	Missense	C	T	0.98	Ala161Val	*RPL8B*
	5	985590	Missense	C	A	1.00	Asn181Lys	*RLM1*

a NA, not applicable.

### Micafungin-resistant isolates carry different *FKS1* mutations.

Between days 6 and 11, seven isolates (D6.1, D7.1, D7.2, D8.1, D8.2, D9.1, and D11.1) acquired increased micafungin MIC values. Six of these isolates carried one of three different nonsynonymous *FKS1* mutations. Fks1, the catalytic portion of 1,3-β-d-glucan synthase, is the target of echinocandin drugs (10). Mutations in *FKS1* are the primary cause of acquired echinocandin resistance and typically occur in one of two small regions, referred to as hot spots, that are well conserved in Candida species (9, 32). Two of the three mutations we identified, *FKS1^R1352C^* and *FKS1^L1355S^*, were in the second hot spot region of the C. lusitaniae *FKS1* gene. One of these hot spot alleles, *FKS1^L1355S^*, was shared by four sequential isolates (D7.1, D7.2, D8.1, and D8.2). The third *FKS1* allele, *FKS1^L685F^*, was located outside both hot spot regions and was carried by the isolate with the highest micafungin MIC value (isolate D11.1, MIC = 1 μg/mL).

### *ERG3* missense mutation is associated with cross-resistance to fluconazole.

Surprisingly, isolate D9.1 had an increased micafungin MIC and was the only fluconazole-resistant and fluconazole-tolerant isolate but had none of the identified *FKS1* mutations. A single coding mutation (Q308K) was identified in *ERG3* in D9.1 that was not present in any other isolate. Importantly, no other unique coding mutations were identified in D9.1. *ERG3* mutations have been reported alone and in conjunction with other resistance mutations in clinical isolates of C. albicans, C. auris, C. lusitaniae, Candida parapsilosis, and C. tropicalis (14, 16, 27, 33–35). These isolates have increased fluconazole resistance and various patterns of resistance to other antifungal drugs and have emerged in patients treated with drugs from multiple antifungal drug classes, including polyenes, echinocandins, and azoles (27, 35). Our results provide the first evidence that acquisition of *ERG3*-mediated drug cross-resistance can occur *in vivo* during echinocandin monotherapy.

### *In vivo* sublineages carry competing adaptive mutations.

**FIG 5:**
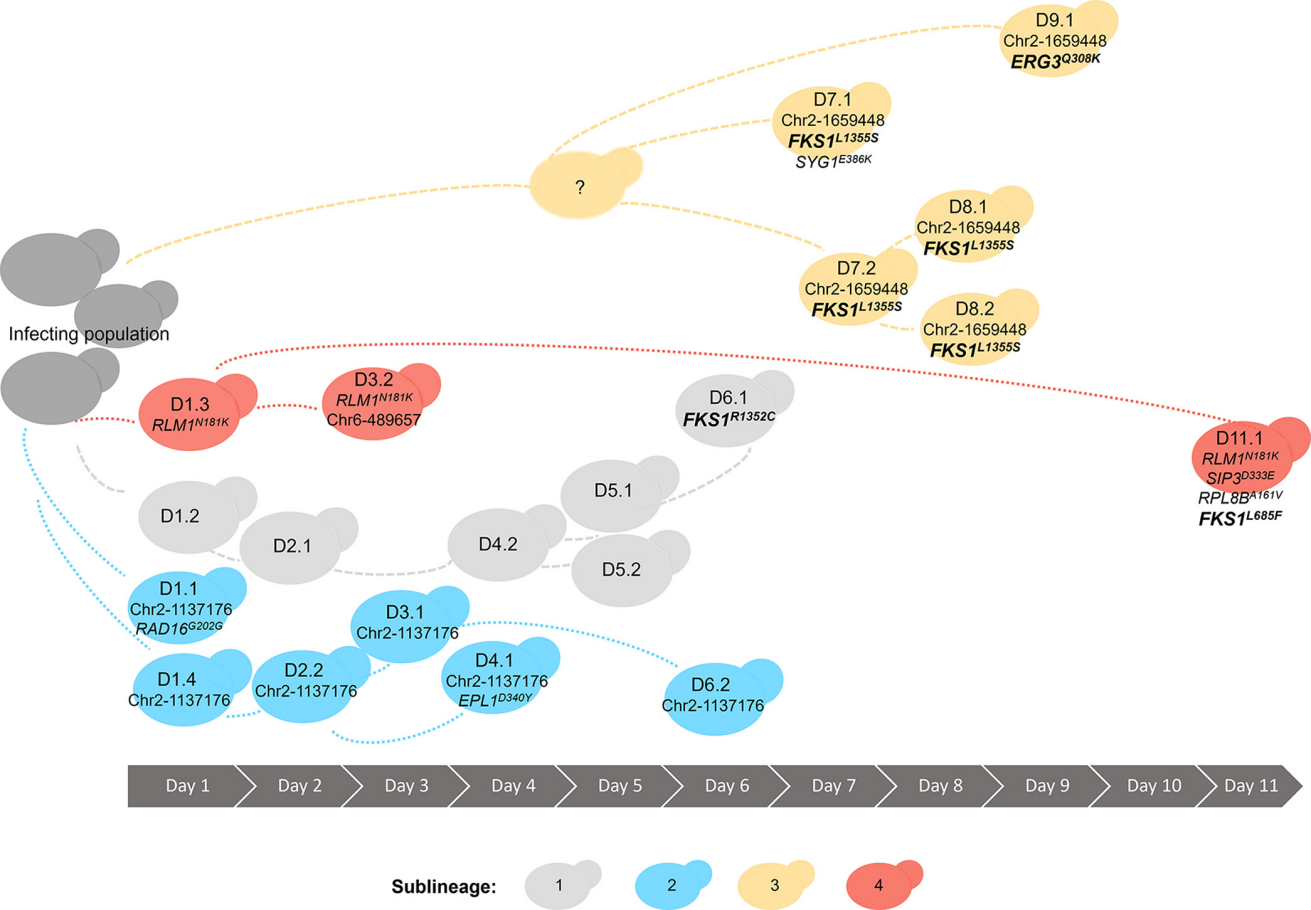
Multiple sublineages with competing mutations evolved *in vivo* during acute C. lusitaniae infection. Representation of four different sublineages identified by shared point mutations and their duration during clinical sampling. Sublineages 2, 3, and 4 included isolates that carried unique mutations in addition to those shared within the sublineage.

The presence of three unique *FKS1* mutations prompted us to further examine the genetic relationships among the sampled isolates. By comparison of all isolate-specific mutation data, we identified four primary sublineages that either had no mutations or shared specific subsets of mutations (Fig. 5; Table S5). The first sublineage was sampled from day 1 to day 6. Five of the six isolates in this sublineage had no interisolate SNPs (isolates D1.2, D2.1, D4.2, D5.1, and D5.2) (Fig. 5, light gray). Isolate D6.1, identified by its acquisition of a single SNP (*FKS1^R1352C^*), is inferred to belong to this originating sublineage (Fig. 5, light gray). A second sublineage, sampled between day 1 and day 6, shared a single intergenic SNP at nucleotide position 1137176 of chromosome 2 (Chr2:1137176) (isolates D1.1, D1.4, D2.2, D3.1, D4.1, and D6.2) (Fig. 5, bright blue). In addition to the shared intergenic SNP, isolate D1.1 acquired a unique synonymous SNP in the ortholog of C. albicans *RAD16* and isolate D4.1 acquired a unique missense SNP in the C. albicans *EPL1* ortholog. The third sublineage was inferred from a shared intergenic SNP at position Chr2:1659448, sampled between days 7 and 9 (Fig. 5, light goldenrod color). No isolates carrying only this intergenic SNP were identified, but it was present in all the isolates with the *FKS1^L1355S^* allele (D7.1 to D8.2), as well as the *ERG3^Q308K^* mutant (D9.1), providing evidence for a shared progenitor prior to divergence and separate acquisition of either the *FKS1^L1355S^* or *ERG3^Q308K^* missense mutation. The fourth sublineage, which shared *RLM1^N181K^*, included isolates D1.3, D3.2, and D11.1 (Fig. 5, dark coral color). In addition to the *RLM1* mutation, isolate D11.1 had acquired the *FKS1^L685F^* allele and missense mutations in *SIP3* and *RPL8B*, indicating that this sublineage persisted throughout the course of the patient’s hospital admission (day 1 to day 11), acquired multiple adaptive mutations, and acquired the highest *in vitro* resistance to the patient’s selected antifungal therapy.

## DISCUSSION

In this study of an individual candidemia case, we have identified the rapid appearance of unsuspected antifungal drug cross-resistance within 7 days of initiating therapy. To our knowledge, this is the clearest genomic study to date of within-patient acquisition of multidrug resistance during antifungal monotherapy. Through our analysis of 20 serial clinical isolates of C. lusitaniae collected during micafungin monotherapy, we identified three unique *FKS1* mutations, including a mutation located outside both hot spot regions of the gene. Analysis of echinocandin-resistant Candida clinical isolates has frequently relied on targeted sequencing of *FKS* hot spot regions (36, 37). However, up to 30% of echinocandin-resistant clinical isolates have no *FKS* mutations identified through this approach (36), and experimental evolution studies have identified non-hot spot mutations in up to 22% of *FKS1* mutants, highlighting the importance of the entire *FKS* gene in echinocandin resistance (38). Importantly, the isolate that obtained the highest micafungin MIC in our study contained a non-hot spot mutation (D11.1, *FKS1^L685F^*) and this isolate did not incur a fitness cost in the absence of drug, unlike the other isolates with *FKS1* hot spot mutations. The probability and effect of all possible *FKS1* mutations on fitness, alone and in combination with other SNPs, will help provide a better understanding of the selection that is occurring during invasive Candida infections.

The identification of multiple, competing *FKS1* mutations within this infection also emphasizes the role of clonal interference in the evolution of antifungal drug resistance during micafungin monotherapy. Clonal interference, the competition of subpopulations carrying different mutations within an asexual population, has been extensively studied in bacteria and Saccharomyces cerevisiae (39, 40). The impact of clonal interference on the acquisition and emergence of drug-resistant mutations in any Candida population, especially within-patient populations, is not known. Our data are limited by the single-colony sampling approach that is standard for clinical testing, and the number of resistance pathways under selection may be underestimated here because of these sampling limitations. Additional approaches, including deep sequencing of whole-blood samples, will be useful in estimating the frequency and dynamics of mutations in the fungal population during acute infections. However, our repeated sampling of the same distinct sublineages over the course of the infection indicates that these sublineages were persistent and represent a significant portion of the infecting population. Our results highlight the within-patient evolution that is occurring during invasive C. lusitaniae infections and underscore the rapid emergence of drug resistance in this species during micafungin monotherapy.

We identified the development of micafungin and fluconazole cross-resistance within 7 days of initiation of echinocandin monotherapy. Cross-resistance was associated with an *ERG3^Q308K^* missense mutation, the only unique coding mutation in this isolate. Resistance to both echinocandins and azoles resulting from *ERG3* missense mutations has been reported in other Candida species. For example, a clinical C. parapsilosis isolate with an *ERG3^G111R^* mutation had reduced azole and echinocandin susceptibility following combination therapy with caspofungin and fluconazole (35). Recently, *in vitro* experimental evolution in the distantly related C. glabrata demonstrated that acquisition of *ERG3* mutations during adaptation to the echinocandin anidulafungin provided cross-resistance to fluconazole (38). Notably, the development of echinocandin-azole cross-resistance depended on the type of *ERG3* mutation acquired—mutations leading to truncated Erg3 proteins in anidulafungin-resistant isolates did not confer fluconazole resistance, while *ERG3* missense mutations resulted in fluconazole cross-resistance (38). Furthermore, the effects of *ERG3* deletion on drug cross-resistance vary by species: C. glabrata cells with an *erg3* deletion are fluconazole susceptible (41), C. albicans cells with homozygous *erg3/erg3* deletions are azole resistant but not echinocandin resistant (35), and C. parapsilosis cells with homozygous *erg3/erg3* deletions are cross-resistant to azoles and echinocandins (35). In C. lusitaniae, the type of *ERG3* mutation also appears to influence the level of azole resistance: a multidrug-resistant clinical isolate with a nonsense mutation at ERG3^Q308^* remained susceptible to fluconazole (27), while the missense mutant *ERG3^Q308K^* reported here is fluconazole resistant. Further work is required to understand the interspecies variability in *ERG3*-mediated antifungal drug resistance and cross-resistance patterns.

In addition to drug resistance, we also analyzed drug tolerance, because tolerance is linked to treatment failures and an inability to clear fungal infections (19, 20, 42). We identified increased tolerance of micafungin and fluconazole, primarily in isolates that also acquired decreased susceptibility to the drugs. For example, fluconazole tolerance was generally low in the susceptible isolates, but the single isolate (D9.1) with an increased fluconazole MIC also had high tolerance. The correlation between MIC and SMG in C. lusitaniae may not be consistent, as the two phenotypes are distinct in C. albicans and influenced by multiple factors, including genetic background and temperature (20, 43). The evolution of drug tolerance *in vitro* is also driven by drug concentration: high concentrations (supra-MICs) of azole drugs select for increased tolerance, whereas low concentrations (MIC and below) select for increased resistance (43, 44). While we do not know the serum levels of micafungin in this patient, it is tempting to speculate that the increased micafungin dosing on day 8 selected for lineages with both increased tolerance and resistance. Ultimately, the high tolerance found in these clinical isolates suggests that even if azoles had been a therapeutic option in this patient, the infection would have persisted.

The results described here demonstrate the value of comparative analysis of serial isolates collected during acute infection. The rapid increase in micafungin MIC values and associated mutations are of significant relevance in clinical care for treatment considerations of Candida bloodstream infections, especially in the setting of recurrent isolation of the same organism over several days. Infectious Diseases Society of America clinical practice guidelines for the treatment of invasive candidiasis currently recommend testing for azole susceptibility for all bloodstream and other clinically relevant Candida isolates. Testing for echinocandin susceptibility is recommended only in patients who have had prior treatment with an echinocandin and among those who have infection with C. glabrata or C. parapsilosis (45). No guidelines exist on frequency of testing. In our clinical practice, in consideration of resource utilization, antifungal susceptibility testing is typically performed only on the first isolate, and subsequent susceptibility tests must be specifically requested by the treating physician. This study demonstrates the significance of reevaluating the standard frequency of antimicrobial susceptibility testing when the same Candida species is detected from blood recurrently. Rapid emergence of resistance also informs antifungal stewardship programs. This is the first clinical report of an *ERG3* mutation in C. lusitaniae associated with resistance to multiple drug classes during echinocandin monotherapy. We hope that an awareness that echinocandin therapy failure can result in fluconazole cross-resistance in C. lusitaniae may improve future therapeutic interventions.

## MATERIALS AND METHODS

### Ethical considerations.

The study was reviewed by the University of Minnesota Institutional Review Board (IRB identification number [ID] STUDY00006473) and was determined to meet the criteria for exemption.

### Clinical antifungal susceptibility testing.

Susceptibility testing of isolate D1.1 was performed by the hospital clinical microbiology laboratory using Sensititre YeastOne broth microdilution plates according to the manufacturer’s instructions.

### Isolates and growth conditions.

Clinical isolate data are provided in Table S1. Each isolate is a single-colony subculture of an individual clinical blood culture. Colonies were cultured on Sabouraud dextrose agar plates, and stocks were prepared with 20% glycerol and stored at −80°C. Overnight cultures were grown in a shaking incubator at 30°C in liquid YPAD medium with 2% dextrose (10 g/L yeast extract, 20 g/L Bacto peptone, 20 g/L dextrose, 0.04 g/L adenine, and 0.08 g/L uridine).

### MIC and SMG testing.

The MIC values for fluconazole (product no. J62015; Alfa Aesar) and micafungin (product no. HY16321; Medchemexpress) were determined by broth microdilution performed in YPAD medium with 1% dextrose as described previously (46). The amphotericin B (product no. 1397-89-3; Chem-Impex International) MIC values were determined by broth microdilution performed in RPMI 1640 medium with 0.2% glucose buffered with 0.165 M MOPS (morpholinepropanesulfonic acid) and adjusted to pH 7.0. Overnight cultures were diluted in fresh medium to a final optical density at 600 nm (OD_600_) of 0.01. Twenty-microliter amounts of the dilution were inoculated into wells of 96-well plates containing 180 μL of 2-fold serial dilutions of antifungal drug. Plates were incubated in a humidified chamber at 30°C, and OD_600_ readings were performed at 24- and 48-h postinoculation in a BioTek Epoch2 plate reader. The European Committee on Antimicrobial Susceptibility Testing (EUCAST) method for susceptibility testing of yeasts defines the MIC for azoles and echinocandins as the lowest drug concentration that inhibits ≥50% of growth relative to the growth of a no-drug control and the amphotericin B MIC as the lowest concentration that inhibits ≥90% of growth relative to the growth of a no-drug control (47). Fluconazole and micafungin MIC_50_ and amphotericin B MIC_90_ values were read at 24 h. Antifungal drug tolerance, quantified as supra-MIC-growth (SMG), was calculated as the mean value for 48-h growth in all wells with drug concentrations above the MIC divided by the mean value for growth in the no-drug wells. All susceptibility assays were performed in triplicate.

### Growth curve analysis.

Overnight cultures were diluted in fresh medium to a final OD_600_ of 0.01, and 10-μL amounts of this cell suspension were inoculated into cells of a 96-well plate containing 190 μL of YPAD medium with 2% dextrose or YPAD medium with 1% dextrose and diluted micafungin (0.016 μg/mL or 0.032 μg/mL). Cells were grown at 30°C in a BioTek Epoch 2 plate reader with shaking for 48 h with OD readings every 15 min. Growth curves were performed in triplicate. Growth curve metrics, including AUC-L, were calculated with the R package *Growthcurver* (version 0.3.1) (21, 48). Isolates were grouped by micafungin MIC value; data were normalized by Box-Cox transformation, and omnibus testing of mean AUC-L was performed using Welch’s analysis of variance (ANOVA) followed by the *post hoc* Games-Howell test, which includes multiple test correction. Statistical analysis was performed with the R packages *MASS* (version 7.3-58.3) and *rstatix* (version 0.7.0) (49, 50).

### Illumina whole-genome sequencing.

Genomic DNA was isolated from overnight cultures using phenol-chloroform extraction as previously described (51). Libraries were prepared using the Illumina DNA prep kit and Integrated DNA Technologies (IDT) 10-bp unique dual indexes (UDIs). Samples were sequenced on an Illumina NextSeq 2000 to produce paired-end 151-bp reads. Bcl-convert (version 3.9.3) was used for demultiplexing, quality control, and adapter trimming (52). Low-quality reads were trimmed with Trimmomatic (version 0.39; options LEADING:3 TRAILING:3 SLIDINGWINDOW:4:15 MINLEN:36 TOPHRED:33) (53).

### Reference genome alignment and variant detection.

To select a reference genome, we chose multiple C. lusitaniae genome assemblies from NCBI and MycoCosm (NCBI accession numbers GCA_000003835.1, GCA_001673695.2, GCA_014636115.1, GCA_009498055.1, and GCA_003675555.2 and MycoCosm MJ12 version 1.0) (23–29). We aligned the trimmed reads of isolate D1.1 to each assembly using the BWA-MEM tool (Burrows-Wheeler Alignment–maximal exact matches; version 0.7.17) with default parameters (54). We sorted aligned reads and marked duplicates using the Samtools (version 1.10) utilities fixmate, sort, and markdup (55). We then generated basic alignment statistics using Samtools flagstat (55). The genome assembly from the FDA-ARGOS microbial sequence database (strain 655; NCBI accession number GCA_014636115.1) had the highest percentage of mapped, properly paired reads and was used as the reference genome for all variant analyses (25).

Small variants (SNPs and small indels) were called using *freebayes* with initial filtering for read depth of at least 10 and alternate-allele frequency of at least 0.9 (56). We used vcftools (version 0.1.16) and bcftools (version 1.10.2) to additionally filter for mapping quality greater than 40, number of observations on the reverse strand greater than 0, number of reads placed to the left/right of the variant greater than 1, strand balance probability of the alternate allele greater than 0, no more than one SNP within 5 bp, and no more than one indel within 10 bp (57, 58). We performed annotation and effect prediction using snpEff (59). The snpEff database was built manually using the FDA-ARGOS reference genome fasta and GFF files and configured for the yeast alternate-codon table. To identify *de novo* mutations among the 20 serial isolates, we used bcftools to filter all fixed variants (variants with allele count equal to number of samples) and visually verified candidate *de novo* variants in IGV (60).

### SNP-based isolate clustering.

Multiple-correspondence analysis was performed for all serial isolates and four unrelated isolates (strain MEC245 from an unrelated patient, strains MEC285 and MEC286 from a second unrelated patient, and strain ATCC 42720, using publicly available sequencing data at GenBank accession number GCA_003675505.1). SNPs were called and filtered for quality and to remove fixed variants as described above. GATK (version 4.1.2) VariantsToTable was used to output SNPs to a tab-delimited table (61). The R package *FactoMineR* (version 2.6) was used to perform MCA using the resulting allele table (62). MCA results were obtained using the R package *ggplot2* (version 3.4.2) (63).

### Copy number analysis and genome-wide visualization.

Copy number variations (CNVs) were analyzed using a read-depth-based approach. We calculated and corrected the GC bias of bam files using deeptools (version 3.5.1) (64). The GC-corrected bam files were used as input to mosdepth (version 0.3.3) to determine the mean depth per 2,000-bp fixed window across the genome (65). For each isolate, the mean per-window read depth was then normalized to the mean read depth of the nuclear genome. We used the normalized results to plot copy number for each isolate using the R package *karyoploteR* (version 1.22.0) (66).

### Data availability.

The sequencing data generated during this study are available in the Sequence Read Archive repository under project accession number PRJNA954073.
