# Supplementary material for: Rapid Evolution of Multidrug Resistance in a Candida lusitaniae Infection during Micafungin Monotherapy
Source: Antimicrob Agents Chemother. 2023 Jul 10;67(8):e00543-23. doi: 10.1128/aac.00543-23 (PMC10433866; doi:10.1128/aac.00543-23)
Supplement: Supplemental file 1 — Supplemental material. Download aac.00543-23-s0001.pdf, PDF file, 0.04 MB [file aac.00543-23-s0001.pdf]

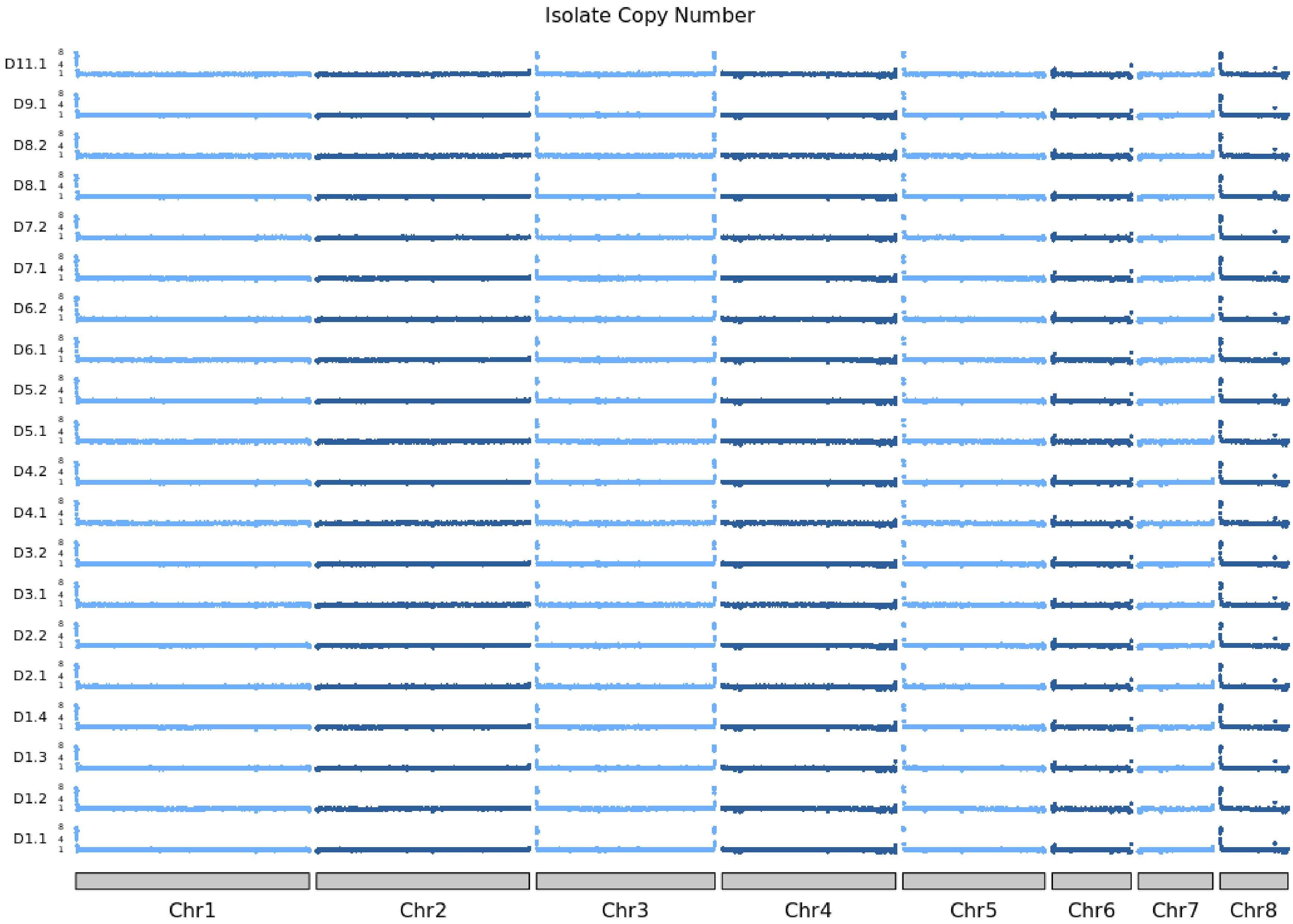

Figure S1. Genome-wide copy number visualization supports the clonal origin of all serial isolates. Plot of normalized read depth across all chromosomes for all 20 serial isolates. Serial isolates are ordered from D1.1 on the bottom to D11.1 on the top. Chromosomes are ordered by size on the x-axis and estimated mean copy number is indicated on the y-axis. All chromosomes are present in 1 copy, with small increases in mean copy number found in all 20 isolates at repetitive loci, predominantly in the sub-telomeric regions.
